# RCT OF A BEHAVIORAL, TELEHEALTH WELLNESS PROMOTION PROGRAM FOR MIDDLE-AGED ADULTS WITH DISABILITIES

**DOI:** 10.1093/geroni/igad104.3802

**Published:** 2023-12-21

**Authors:** Ivan Molton, Donovan Rivera, Katie Singsank, Aaron Flaster, Leah Munroe, Andrew Humbert

**Affiliations:** University of Washington, Seattle, Washington, United States; University of Washington, Seattle, Washington, United States; University of Nevada, Las Vegas, Las Vegas, Nevada, United States; University of Washington, Seattle, Washington, United States; Louisiana State University, Baton Rouge, Louisiana, United States; University of Washington, Seattle, Washington, United States

## Abstract

For people aging with physical disabilities, there is a striking need for evidence-based approaches to promote positive health behaviors and to support active community participation. This is especially true in midlife, which is a period of significant health and social vulnerability for this population. This study describes a 3-arm, randomized controlled trial of a telehealth, motivational-interviewing based health promotion program (EnhanceWellness, EW) adapted for people with physical disability. 505 individuals with long-term physical disability were randomized to 8 sessions of individual EW coaching over 6 months, or to one of two control conditions (health education or treatment as usual). Primary outcomes included self-report of ability to participate in social activities, satisfaction with community participation, and mood, using validated NIH PROMIS scales. Participants completed assessments before randomization, at 7 and 12 months post-randomization. Statistical analysis included linear mixed-effects modeling with random intercepts. Participants were on average 56 years old (SD=5.6), were mostly women (77%) and had lived with a medical condition causing ADL impairment (disability) for an average of 23 years. Results demonstrated significant group by time interaction terms (p’s < .01), underlying statistically significant improvements in the EW group in both ability to participate and satisfaction with community participation, relative to either control condition. These changes tended to increase or maintain over time. There were no significant intervention effects on mental health outcomes. Results emphasize the applicability of individualized health behavior change coaching in supporting community participation among adults aging with disability.

